# Poster Session II - A241 WHAT IS THE CURRENT LANDSCAPE OF IBD NURSING IN CANADA AND ARE THERE ENOUGH NURSES TO HELP CARE FOR PERSONS WITH IBD?

**DOI:** 10.1093/jcag/gwaf042.241

**Published:** 2026-02-13

**Authors:** N Rohatinsky, A Hoffinger, H Li, A Carter, U Chauhan, B Currie, M Martin, M Watson

**Affiliations:** Nursing, University of Saskatchewan, Saskatoon, SK, Canada; Nursing, University of Saskatchewan, Saskatoon, SK, Canada; Nursing, University of Saskatchewan, Saskatoon, SK, Canada; Oshawa Clinic Group, Oshawa, ON, Canada; McMaster Medical Centre, Hamilton, ON, Canada; QEII Health Sciences Centre, Halifax, NS, Canada; University of Calgary, Calgary, AB, Canada; London Health Sciences Centre, London, ON, Canada

## Abstract

**Background:**

The last survey of Canadian IBD nurses occurred more than 12 years ago. At that time, there were approximately 79 nurses who provided IBD related care. Nurses are key members of multidisciplinary IBD care team promoting the biopsychosocial health of persons living with IBD. Better health outcomes occur when nurses are part of the care team, however, sustainable funding for IBD nurse positions across Canada is a challenge. It is important to determine if the numbers of IBD nurses in Canada are increasing, how nurses’ roles and responsibilities have changed over time, and if nurses are being provided with IBD-related educational opportunities to comprehensively care for persons with IBD.

**Aims:**

The purpose of this quality improvement project, organized by the Canadian IBD nurses (CANIBD) organization, was to determine the number of IBD nurses across Canada, identify their roles and responsibilities, understand how their positions were funded, and identify the available educational opportunities for them.

**Methods:**

A descriptive cross-sectional survey was shared with Canadian IBD nurses between November 2023 and August 2024 via SurveyMonkey.

**Results:**

97 Canadian IBD nurses completed the survey. 56.7% (n = 55) were 45 years of age and younger and the majority were female (n = 94, 96.9%). Most nurses worked in Ontario (n = 52, 53.6%) and provided care to adults with IBD (n = 66, 68%). 27.8% (n = 27) had an advanced degree. 42.3% (n = 41) of nurses had more than 20 years of nursing experience, but 64.9% (n = 63) had less than 10 years in gastroenterology. Nurses’ primary employment setting was hospital outpatient care (n = 38, 39.2%). Nursing positions were primarily funded through hospitals (n = 40, 41.2%) or pharmaceutical companies (n = 26, 26.8%). Nurses provided multiple services including outpatient services (n = 71, 73.2%), telephone advice lines (n = 47, 48.5%), and management of biological therapy services (n = 46, 47.4%). Education to other staff related to IBD was provided by nurses (n = 45, 46.4%). Nurses identified that employers supported learning (n = 83, 85.6%).

**Conclusions:**

Although the numbers of IBD nurses have risen slightly in the past 12 years, the gain is relatively modest. More IBD nurses are needed to help care for persons with IBD given the rising prevalence of IBD in Canada. Further work must be done to secure funding for IBD nurse positions, recruit nurses into the IBD specialty, and to provide nurses with IBD-related education to address the care needs of persons living with IBD.

**Funding Agencies:**

None

